# The Endoscope

**Published:** 1867-08

**Authors:** 


### *The Endoscope.*

The lamented death of Dr. Hunt, noticed on another page, will not interfere with the completion of the work he was engaged in translating for THE JOURNAL. Arrangements have been made that the concluding pages shall be furnished without delay.

